# Using real-world data to estimate the changing trends in the prevalence and incidence of type 2 diabetes mellitus in Xiamen of China from 2014 to 2019

**DOI:** 10.1186/s12902-021-00759-w

**Published:** 2021-05-01

**Authors:** Weiwei He, Qiushi Xu, Lili Han, Ting Wu, Xiulin Shi, Lishan Ye, Guanhua Yao, Xuejun Li

**Affiliations:** 1 School of Medicine, Xiamen University, Xiamen, 361003 China; 2 Xiamen Diabetes Institute, The First Affiliated Hospital of Xiamen University, Xiamen, 361003 China; 3 Fujian Provincial Key Laboratory of Translational Medicine for Diabetes, Xiamen, 361003 China; 4 Xiamen Health and Medical Big Data Center, Xiamen, China; 5 Fujian Medical University, Fuzhou, Fujian China; 6 Department of Endocrinology and Diabetes, The First Affiliated Hospital of Xiamen University, No.55 Zhenhai Road, Xiamen, 361003 China; 7 Xiamen Municipal Health Commission, Xiamen, China

**Keywords:** Type 2 diabetes mellitus, Prevalence, Incidence, Xiamen

## Abstract

**Background:**

The prevalence of diabetes is increasing worldwide. Our study aimed to estimate the changing trends in the prevalence and incidence of diagnosed type 2 diabetes mellitus (T2DM) among Xiamen residents and the floating population using real-world data.

**Method:**

We used real-world data from the System of Xiamen Citizens Health Information from 2014 to 2019 to estimate the changing trends in the prevalence and incidence of diagnosed T2DM. The System included the diagnosis of diabetes and the prescription of hypoglycemic drugs. Prevalent cases of T2DM were individuals who were diagnosed with T2DM and/or using hypoglycemic drugs. Incident cases were individuals with diagnosed T2DM and/or using hypoglycemic drugs in 2014 or 2019 who had not been diagnosed and/or did not use hypoglycemic drugs in the past.

**Results:**

In 2014 and 2019, the prevalence of T2DM in Xiamen was 4.04 and 4.84%, respectively. In 2014 and 2019, the incidence rate of T2DM in Xiamen was 14.1 per 1000 person-year and 15.0 per 1000 person-year, respectively. There was a significant increase in both the prevalence (Prevalence difference: 0.80, 95%CI 0.76–0.83%, *P* < 0.001) and the incidence of T2DM (Incidence difference: 0.9, 95%CI 0.7–1.1, *P* < 0.001). in Xiamen. The prevalence and incidence of T2DM in people aged 18–39 increased significantly (*P* < 0.001), while the prevalence and incidence of T2DM in people aged 40–69 reduced significantly (*P* < 0.001).

**Conclusions:**

There was a significant increase in the prevalence and incidence of T2DM in Xiamen from 2014 to 2019 especially among those with younger age.

## Background

Diabetes is a chronic and progressive disease caused by genetic and environmental factors that can lead to a chronic damage, dysfunction, and even failure of multiple organs [1]. Owing to its high prevalence and high risk of disability, diabetes has become a serious health problem worldwide [2, 3]. With the rapid development of the economy, the improvement of people’s living standards, and the change of lifestyle in China in the past three decades, diabetes has become a chronic disease that seriously endangers people’s health [4]. Studies in recent years have shown that the global prevalence of diabetes is significantly increased and varies by age, geography, regional economic disparities, ethnicity, etc. [5].

Over the past 40 years, the prevalence of diabetes in China has increased from less than 1 to 12.8% in 2018, and the prevalence of prediabetes is 35.2%, making it the country with the largest number of diabetes cases in the world [2, 6]. The total prevalence of diabetes in China from 2007 to 2008 was 9.7%, among which the prevalence of diagnosed diabetes was 4.1% in males and 3.5% in females [7]. The overall prevalence of diabetes in 2010 in China was 11.6%, with the prevalence of diagnosed diabetes at 3.5% [5]. The overall prevalence of diabetes in 2013 in China was 10.9%, of which the prevalence of diagnosed diabetes was 4.0% (3.9% in males, 4.1% in females) [6].

Studies have revealed that the prevalence and the number of adults with diabetes have increased more in low- and middle-income countries than in middle- and high-income countries [8]. Rapid economic development and urbanization have resulted in an increasing burden of diabetes in many parts of the world [9]. As an important central city on the southeast coast of China, Xiamen’s urbanization has reached up to 89.2%. The data from the Xiamen Government Finance Bureau have shown that diabetes-related costs in Xiamen exceeded 43 million dollars in 2019, accounting for approximately 14.2% of the total annual cost of medical care. In terms of health expenditure, the global annual health expenditure on diabetes is estimated at 760 billion dollars. By 2030, spending in this area is expected to reach 825 billion dollars [10]. The cost of diabetes care is statistically at least 3.2 times higher than the per capita medical expenditure and up to 9.4 times higher in the case of diabetes-related complications, so the impact on individuals and society at large if the development of diabetes is not controlled promptly is incalculable [11, 12]. Therefore, exploring the prevalence and incidence of diabetes in Xiamen is of great significance to the management and China implemented healthcare reforms of diabetes in coastal areas of China. However, there are few data on the prevalence and incidence of diabetes in Xiamen. In this study, we used real-world data from the System of Xiamen Citizen Health Information to estimate the changing trends in the prevalence and incidence of diagnosed type 2 diabetes mellitus (T2DM) among Xiamen residents and the floating population.

## Methods

### Health system

The data of this study were from the System of Xiamen Citizens Health Information, which is comprehensive information management and service platform jointly built by the Xiamen Municipal Health Bureau and China Mobile. All patients have a unique health insurance number and include information from all medical records of community hospitals, secondary and tertiary hospitals, and pharmacies, as well as diagnosis and medication information related to diabetes.

### Data collection

In this study, the real-world data from the System of Xiamen Citizens Health Information included the diagnosis of diabetes and usage records of hypoglycemic drugs such as Biguanides, Sulfonylureas, Thiazolidinediones and DPP-4 inhibitors. Prevalent cases of T2DM were individuals who were diagnosed with T2DM and/or used hypoglycemic drugs. To estimate the prevalence of T2DM in 2014 and 2019, we used data of System from 2014 and 2019, respectively. Incident cases were individuals with diagnosed T2DM and/or using hypoglycemic drugs in 2014 or 2019 who had not been diagnosed and/or no used one in the past. Incident cases were individuals with diagnosed T2DM and/or using hypoglycemic drugs in 2014 or 2019 who had not been diagnosed and/or did not use hypoglycemic drugs in the past.

### Statistical analysis

We counted the number of people in the System who met the T2DM definition and used this as a numerator. Population denominator data for the years 2014 and 2019 were population estimates derived by the government based on the 2013 and 2018 censuses, respectively. When calculating the prevalence of T2DM in Xiamen’s floating population in 2019, the denominator was based on the data of the floating population in Xiamen released by the government in 2018. In this study, all included T2DM patients were analyzed according to gender and age, the age groups were 18–29 years, 30–39 years, 40–49 years, 50–59 years, 60–69 years, and over 70 years. The information of each subgroup in 2019 was compared with that of 2014, and statistical analysis was carried out using SPSS18.0 and χ2 test, with *P*<0.05 indicating that the difference was statistically significant.

## Results

### Prevalence of T2DM in Xiamen

**Fig. 1:**
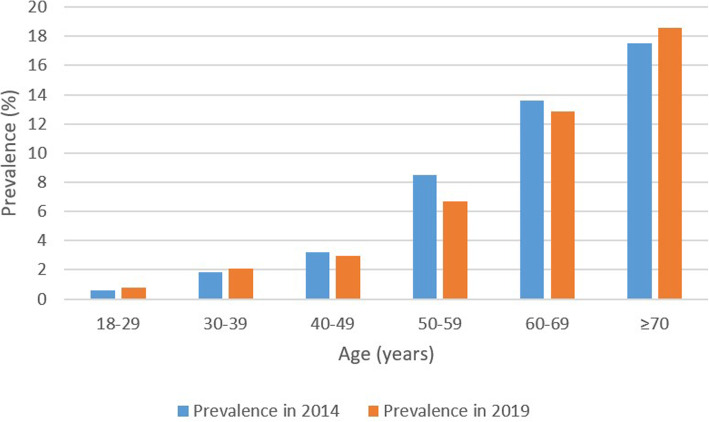
Prevalence of type 2 diabetes mellitus among populations with different ages in 2014 and 2019

In 2014, the resident population of Xiamen was 2,932,637 and the number of T2DM patients was 118,468, with a prevalence of 4.04%. The prevalence of T2DM increased with age, and it grows rapidly after the age of 40 years old. The number of patients aged 50–59 years old was the largest, with a prevalence of 8.47% (95%CI 8.38–8.56). The prevalence was as high as 17.54% (95%CI 17.33–17.74) in people over 70 years old. The prevalence of T2DM was 0.60% (95%CI, 0.58–0.62) among 18–29 years old (Table 1; Fig. 1).

**Table 1:** Prevalence rates of type 2 diabetes in 2014 and 2019

	2014 population			2019 population				
	No. of diabetes	General	Prevalence (%) (95%CI)	No. of diabetes	General	Prevalence (%) (95%CI)	Prevalence difference (95% CI)	***P*** value
Total	118,468	2,932,637	4.04 (4.02–4.06)	140,535	2,905,138	4.84 (4.81–4.86)	0.80 (0.76–0.83)	< 0.001
Sex								
Females	68,641	1,455,557	4.72 (4.68–4.75)	78,553	1,423,444	5.52 (5.48–5.56)	0.80 (0.75–0.85)	< 0.001
Males	49,787	1,477,080	3.37 (3.34–3.40)	61,982	1,481,694	4.18 (4.15–4.22)	0.81 (0.77–0.86)	< 0.001
Age, Years								
18–29	5143	857,876	0.60 (0.58–0.62)	4141	530,836	0.78 (0.76–0.80)	0.18 (0.15–0.21)	< 0.001
30–39	14,378	775,476	1.85 (1.82–1.88)	17,594	836,915	2.10 (2.07–2.13)	0.25 (0.21–0.29)	< 0.001
40–49	19,945	620,726	3.21 (3.17–3.26)	18,513	627,754	2.95 (2.91–2.99)	− 0.26 (− 0.33 ~ − 0.20)	< 0.001
50–59	29,073	343,264	8.47 (8.38–8.56)	29,840	447,403	6.67 (6.60–6.74)	−1.80 (− 1.92 ~ − 1.68)	< 0.001
60–69	26,348	194,072	13.58 (13.42–13.73)	34,804	270,315	12.88 (12.75–13.00)	− 0.70 (− 0.90 ~ − 0.50)	< 0.001
≥ 70	23,581	134,473	17.54 (17.33–17.74)	35,643	191,915	18.57 (18.40–18.75)	1.04 (0.77 ~ 1.30)	< 0.001

(95%CI, 95% confidence interval)

In 2019, the resident population of Xiamen was 2,905,138, with a total of 140,535 T2DM patients, with a prevalence of 4.84% (95%CI 4.81–4.86). The prevalence of T2DM among women was also higher than men, with 5.52% (95%CI 5.48–5.56) and 4.18% (95%CI 4.15–4.22), respectively. Among the patients, 35,643 patients over the age of 70 years had the most cases of T2DM, with a prevalence of 18.57% (95%CI 18.40–18.75). The prevalence of T2DM was 0.78% (95%CI 0.76–0.80) among the age of 18–29 years old. The prevalence of T2DM increased with age in both men and women and increased sharply after the age of 50 years old.

### The change in the prevalence of T2DM in Xiamen

There was a significant increase in the prevalence of T2DM from 2014 to 2019, and the difference in the increase was 0.80% (95%CI,0.76–0.83%). The increase from 2014 to 2019 was 0.18% (95%CI,0.15–0.21%) in the age of 18–29 years old, 0.25% (95%CI,0.21–0.29%) in the age of 30–39 years old, and 1.04% (0.77–1.30%) in the age of over 70 years old. There was a reduction from 2014 to 2019 among the age of 40–69 years old. The reduction was 0.26% (95%CI,0.20–0.33%) in the age of 40–49 years old, 1.80% (95%CI,1.68–1.92%) in the age of 50–59 years old, and 0.70 (95%CI, 0.50–0.90%) in the age of 60–69 years old (Table 1; Fig. 1).

### Incidence rate of T2DM in Xiamen

**Fig. 2:**
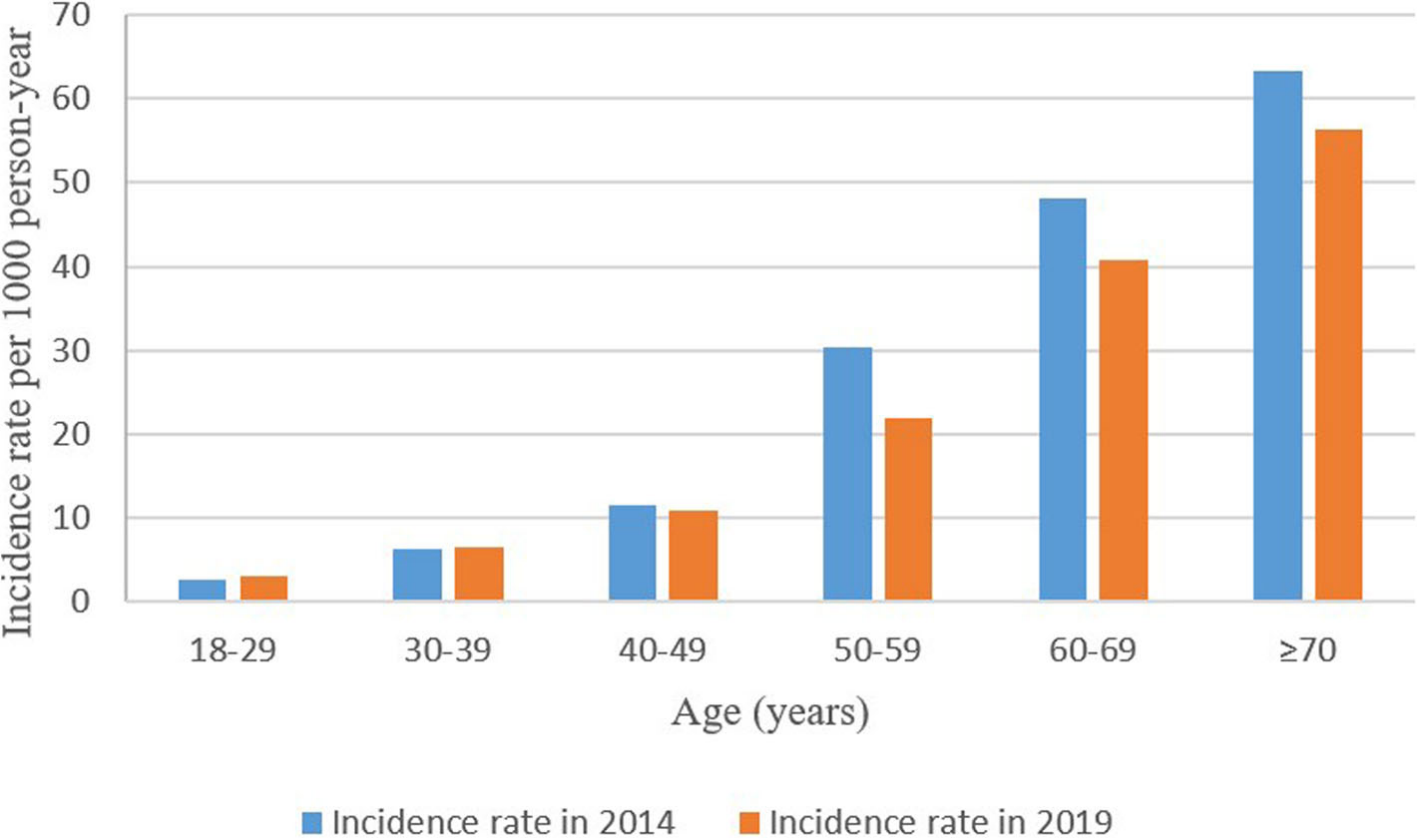
Incidence rate of type 2 diabetes mellitus among populations with different ages in 2014 and 2019

In 2014, the incidence rate of T2DM in Xiamen was 14.1 per 1000 (95%CI, 13.9–14.2), and the incidence rate of females was higher than that of men, which was 15.9 per 1000 (95%CI, 15.7–16.1) and 11.6 per 1000 (95%CI, 11.4–11.8), respectively. The number of newly diagnosed T2DM patients aged 50–59 years old was the largest, and the incidence rate was 30.4 per 1000 (95%CI, 29.8–31.0). The highest incidence rate of new cases was 63.3 per 1000 (95%CI, 61.9–64.7) occurred at the age of over 70 years old. The incidence rate of T2DM was 2.6 per 1000 (95%CI, 2.5–2.7) at the age of 18–29 years old (Table 2; Fig. 2).

**Table 2:** Incidence rates of type 2 diabetes in 2014 and 2019

	2014 population			2019 population				
	No. of diabetes	Total person-year	Incidence rate, per 1000 person-year	No. of diabetes	Total person-year	Incidence rate, per 1000 person-year	Rate difference (95% CI)	***P*** value
Total	39,947	2,843,850	14.1 (13.9–14.2)	41,663	2,780,967	15.0 (14.8–15.1)	0.9 (0.7–1.1)	< 0.001
Sex								
Females	22,274	1,404,400	15.9 (15.7–16.1)	22,585	1,355,475	16.7 (16.5–16.9)	0.8 (0.5–1.1)	< 0.001
Males	16,673	1,439,450	11.6 (11.4–11.8)	19,078	1,425,492	13.4 (13.2–13.6)	1.8 (1.5–2.1)	< 0.001
Age, Years								
18–29	2253	854,868	2.6 (2.5–2.7)	1617	528,056	3.1 (2.9–3.2)	0.4 (0.2–0.6)	< 0.001
30–39	4932	765,511	6.4 (6.3–6.6)	5455	823,436	6.6 (6.5–6.8)	0.2(−0.1 ~ 0.4)	0.155
40–49	6980	606,211	11.5 (11.3–11.8)	6664	610,178	10.9 (10.7–11.2)	−0.6(−1.0 ~ − 0.2)	0.002
50–59	9758	321,105	30.4 (29.8–31.0)	9210	421,281	21.9 (21.4–22.3)	−8.5 (−9.3 ~ −7.8)	< 0.001
60–69	8698	180,487	48.2 (47.2–49.2)	9732	238,499	40.8 (40.0–41.6)	−7.4(−8.7 ~ −6.1)	< 0.001
≥ 70	7326	115,678	63.3 (61.9–64.7)	8985	159,517	56.3 (55.2–57.5)	−7.0 (−8.8 ~ −5.2)	< 0.001

In 2019, the incidence rate of T2DM was 15.0 per 1000 (95%CI, 14.8–15.1), and the incidence rate of females was still higher than that of males, which was 16.7 per 1000 (95%CI, 16.5–16.9) and 13.4 per 1000 (95%CI, 13.2–13.6), respectively. The number of newly diagnosed T2DM patients aged 60–69 years old was 9732, with an incidence rate of 40.8 per 1000 (95%CI, 40.0–41.6). The incidence rate of newly diagnosed T2DM patients over the age of 70 years old was the highest in the same year, as high as 56.3 per 1000 (95%CI, 55.2–57.5). The incidence rate of T2DM increased with age in both men and women and increased sharply after the age of 50 years old.

### The change in the incidence rate of T2DM in Xiamen

There was an increase in the incidence of T2DM from 2014 to 2019, and the difference in the incidence rate was 0.9% (95%CI, 0.7–1.1). The increase from 2014 to 2019 was 0.4% (95%CI, 0.2–0.6) in the age of 18–29 years old. There was a reduction from 2014 to 2019 over the age of 40 years old. The reduction was 0.6% (95%CI, 0.2 ~ 1.0) in the age of 40–49 years old, 8.5% (95%CI, 7.8 ~ 9.3) in the age of 50–59 years old, 7.4% (95%CI, 6.1 ~ 8.7) in the age of 60–69 years old, and 7.0% (95%CI, 5.2 ~ 8.8) over the age of 70 years old (Table 2; Fig. 2).

### The prevalence of T2DM among the floating population

In 2019, there were 840,443 floating population in Xiamen, of which 7933 were diagnosed with T2DM, with a prevalence of 0.94% (95% CI 0.92–0.97). Among them, the age of 30–39 years old was the largest population with T2DM, and the prevalence was 1.13% (95%CI 1.09–1.17). The prevalence of T2DM over 70 years old was the highest, as high as 2.64% (95%CI 2.26–3.01) (Table 3).

**Table 3:** Prevalence of type 2 diabetes in the floating population in 2019

	No. of diabetes	Population	Estimate (%)	95% CI
Total	7933	840,443	0.94	0.92–0.97
Sex				
Females	5203	367,754	1.42	1.38–1.45
Males	2730	472,689	0.58	0.56–0.60
Age, years				
18–29	1388	307,385	0.45	0.43–0.48
30–39	3010	265,476	1.13	1.09–1.17
40–49	1589	143,763	1.11	1.05–1.16
50–59	1229	86,816	1.42	1.34–1.49
60–69	532	30,017	1.77	1.62–1.92
≥ 70	185	7016	2.64	2.26–3.01

## Discussion

In this study, we found that there was a significant increase in the prevalence and incidence rate of T2DM in Xiamen from 2014 to 2019. The prevalence and incidence rate of T2DM in people aged 18–29 and 30–39 years old increased significantly from 2014 to 2019, while the prevalence and incidence rate of T2DM in people aged 40–69 years old reduced from 2014 to 2019, indicating that the T2DM patients in Xiamen showed a trend of younger age. The result of our study was consistent with the concept that diabetes tends to be greater among the young in Asia [13]. The results mentioned above showed that one in five adult patients was diagnosed with T2DM before the age of 40 years old and those younger patients with T2DM had poor awareness of disease management, suggesting that more young people will suffer from T2DM in the future [13]. More focused efforts are needed to improve risk factor control strategies for people under the age of 40 years, including prevention measures for obesity and inadequate lifestyles, and younger screening for diabetes for those persons at risk. Compared with the prevalence of T2DM in 2014, the rapid increase in prevalence in 2019 is closely related to the economic development of Xiamen in recent years, the improvement of people’s living standards, the acceleration of urbanization, and the aging population, etc. With the development of the economy, people’s lifestyles such as dietary structure have undergone major changes, the proportion of cereal intake has decreased, the proportion of meat and fat has increased, fat intake has increased, physical exercise has decreased, all of these can promote the occurrence and development of overweight, obesity, and diabetes [14–16]. This shows that the situation of prevention and treatment of diabetes in Xiamen needs to be taken seriously.

The results of our study found that the prevalence of diagnosed T2DM in Xiamen was 4.84% in 2019. The prevalence among men and women was 4.18 and 5.52%, respectively, which were higher than the prevalence of diagnosed diabetes in 2007, 2010, and 2013 in China. A large national study in mainland China published recently showed that 11.2% of adults (according to WHO standards) or 12.8% of adults (according to ADA standards) had diabetes in 2018, as a result, China was estimated to be 129.8 million persons with diabetes (70.4 million males and 59.4 million females), of which the prevalence of diagnosed diabetes was 6.0% (6.4% in men, 5.6% in women) [17]. The overall prevalence of diabetes is higher in urban than rural areas, but the gap between urban and rural areas is narrowing year by year. The survey concluded that the prevalence of diabetes among adults living in China increased slightly from 2007 to 2017 and that diabetes is an important public health problem in China that requires ongoing surveillance and effective control to reduce its burden. The prevalence of diabetes continues to increase globally, and the trend in diabetes prevalence in China is consistent with that worldwide. Compared to Europe, Chinese patients with T2DM show a younger trend and a lower BMI [18]. .According to the study, the prevalence of diagnosed diabetes in urban and southern China was 7.1 and 5.8% respectively, both higher than the prevalence of T2DM in Xiamen in our study. It has been reported that the incidence of diagnosed T2DM in Beijing decreased from 24.3 (95%CI, 24.2, 24.4) per 1000 person-years in 2008 to 11.5 (95%CI, 11.5, 11.6) per 1000 person-years in 2017, while our study showed that the incidence of diagnosed T2DM increased significantly in 2019 compared with 2014. Consistent with their findings was a higher prevalence and incidence in women than in men [19]. The lower prevalence and incidence rate of T2DM in Xiamen compared to the studies mentioned above may be attributed to many factors. There is considerable evidence that Western diets characterized by meat are significantly associated with T2DM, while Mediterranean diets characterized by plants are associated with a low risk of diabetes [20, 21]. A meta-analysis has concluded that intake of red meat and processed meat is positively associated with the risk of T2DM, while intake of aquatic products has no significant correlation with the risk of T2DM [22]. Our study showed that the prevalence of diagnosed T2DM in Xiamen was lower than the prevalence of diagnosed diabetes in China in 2018 (4.84% vs 6.0%), which may be related to the fact that Xiamen is located in the coastal area of China, where the seafood is abundant and diverse, and the per capita daily intake of aquatic products of Xiamen residents is higher than the average level of all over the world.

The prevalence of diabetes among the floating population is lower than that of the general population, but considering the growth rate of the floating population and the poor disease management awareness among the floating population, the potential threat posed by diabetes among this group cannot be ignored [23–26]. In 2017, a study calculated that the prevalence of diabetes among the floating population in China was 5.1% (95%CI 4.9–5.3), and the prevalence was higher among women than men [25]. Xiamen is a developed coastal economic region with a large migrant population, but there are few reports on the prevalence of diabetes among the floating population. This study explored this issue for the first time and found that in 2019, the number floating population in Xiamen was 840,443, and the prevalence of T2DM was 0.94%(1.42% in women, 0.58% in men). The number of aged 18–29 years old persons was the most floating population, with a prevalence of 0.45%. Those persons over 70 years old have the highest T2DM prevalence, which was 2.64%. It can be seen from the above data that the prevalence of diabetes among the floating population in Xiamen is far lower than the national average level, which may be attributed to the following reasons: 1) Most of the floating population in Xiamen are working people with good physical fitness and great exercise intensity, and there are fewer obese or overweight people, thus reducing the risk of diabetes. 2) Most of the floating population who come to Xiamen does not have medical insurance in Xiamen, so it may not be possible to record diagnosed diabetes cases into the System of Xiamen citizen health information. Overall, the government and the hospital should offer T2DM knowledge lectures or online classes for the floating population in Xiamen, to improve their self-management awareness of T2DM, and effectively control the epidemic trend of T2DM in the floating population.

The strength of our study included a large sample of a population-based longitudinal study to direct comparison of T2DM prevalence and incidence from 2014 to 2019 in the population in Xiamen. The prevalence and incidence rate of T2DM in this study were evaluated based on real and reliable data. There were also several limitations to our study. Firstly, the study did not analyze patients with undiagnosed diabetes who had T2DM but did not receive standard diabetes care or hypoglycemic medications. However, Xiamen is a city with universal health insurance coverage and has relatively good medical coverage compared to other cities in China. Therefore, the current study design can provide a relatively accurate assessment of the prevalence or epidemiology of diabetes in Xiamen. Secondly, the types of hypoglycemic drugs used by patients with diabetes in Xiamen are diverse, and many patients have changed their hypoglycemic drugs frequently, which make it difficult to determine the types of hypoglycemic drugs they commonly use and the changes in the medication use over time. The use of hypoglycemic medications among persons with diabetes in Xiamen and the changes in medication use over time need to be explored in future researches. Thirdly, the prevalence and incidence of T2DM among women in Xiamen were higher than that among men in Xiamen. The clinical visit rates among women in Xiamen are higher than that among men, which is a possible reason for the higher prevalence and incidence of T2DM among women in Xiamen. Finally, we only analyzed T2DM and had no information on other types of diabetes including type 1 diabetes. It is true that some subjects are poorly accessible to medical services, and this issue is indeed a limitation of our study design that may affect the accuracy of the results or data of this study.

## Conclusion

In conclusion, our study used real data from the System of Xiamen Citizen Health Information to estimate trends in the prevalence and incidence of T2DM in Xiamen. We found that the prevalence and incidence rate of T2DM in Xiamen showed a significant increase from 2014 to 2019, and it was more severe among younger people. Through the System of Xiamen Citizens Health Information, the purpose of real-time monitoring trends in prevalence and incidence of T2DM can be achieved, thereby providing a basis for health policies and health service plans.

## Data Availability

The datasets generated or analyzed during the current study are not publicly available due to data sharing policies but are available from the corresponding author on reasonable request.

## Abbreviations

T2DM: Type 2 diabetes mellitus
CI: Confidence interval
